# Intraocular Viral Communities Associated With Post-fever Retinitis

**DOI:** 10.3389/fmed.2021.724195

**Published:** 2021-11-19

**Authors:** Kotakonda Arunasri, Gumpili Sai Prashanthi, Mudit Tyagi, Rajeev R. Pappuru, Sisinthy Shivaji

**Affiliations:** ^1^Brien Holden Eye Research Centre, L. V. Prasad Eye Institute, Hyderabad, India; ^2^Smt. Kanuri Santhamma Center for Vitreo Retinal Diseases, L. V. Prasad Eye Institute, Hyderabad, India

**Keywords:** metagenomic sequencing, vitreous fluid, post-fever retinitis, changes in virome, ocular pathogen, human viruses

## Abstract

The virome of ocular fluids is naive. The results of this study highlight the virome in the vitreous fluid of the eye of individuals without any ocular infection and compare it with the virome of the vitreous fluid of individuals with retinitis. A total of 1,016,037 viral reads were generated from 25 vitreous fluid samples comprising control and post-fever retinitis (PFR) samples. The top 10 viral families in the vitreous fluids comprised of *Myoviridae, Siphoviridae, Phycodnaviridae, Herpesviridae, Poxviridae, Iridoviridae, Podoviridae, Retroviridae, Baculoviridae*, and *Flaviviridae*. Principal coordinate analysis and heat map analysis clearly discriminated the virome of the vitreous fluid of the controls from that of the PFR virome. The abundance of 10 viral genera increased significantly in the vitreous fluid virome of the post-fever retinitis group compared with the control group. Genus *Lymphocryptovirus*, comprising the human pathogen Epstein-Barr virus (EBV) that is also implicated in ocular infections was significantly abundant in eight out of the nine vitreous fluid viromes of post-fever retinitis group samples compared with the control viromes. Human viruses, such as *Hepacivirus, Circovirus*, and *Kobuvirus*, were also significantly increased in abundance in the vitreous fluid viromes of post-fever retinitis group samples compared with the control viromes. The Kyoto Encyclopedia of Genes and Genomes (KEGG) functional analysis and the network analysis depicted an increase in the immune response by the host in the post-fever retinitis group compared with the control group. All together, the results of the study indicate changes in the virome in the vitreous fluid of patients with the post-fever retinitis group compared to the control group.

## Introduction

Genetically diverse groups of viruses having DNA or RNA as their genome can infect eyes and cause different ocular diseases. Viruses may enter the eye by direct contact or *via* haematogenous or neuronal spread (1), leading to viral infections like blepharitis (2), conjunctivitis (3), keratitis (4), uveitis (5), cataract (6), and retinitis (7). Common ocular viral pathogens include Herpes viruses, such as herpes simplex virus (HSV), herpes zoster virus (HZV)/varicella, cytomegalovirus (CMV), Epstein–Barr virus, adenovirus, and vaccinia, which cause epithelial or stromal keratitis, conjunctivitis, etc. (1). Apart from these direct viral infections of the eye, studies have also indicated that several systemic viral infectious agents, such as influenza virus (8), dengue virus (9), chikungunya virus (9), and Zika virus (9), were also found to disseminate into the retina and cause ocular diseases. In contrast to these typical cases of systemic viral infections, at times, a diagnostic dilemma is presented in atypical cases of viral manifestation. The atypical viral presentation may be due to latent virus of a systemic illness that does not cause any pathology for some time after infection, but becomes pathogenic under immune-compromised and immune-suppressed conditions in healthy individuals. Post-fever retinitis (PFR) is one such condition of the retina in which retinitis was observed systematically 2–4 weeks post-febrile illness (10–12). On most occasions, it would be difficult to identify the causative organisms associated with post-fever retinitis. In our earlier study, using the next generation sequencing (NGS) approach, changes in bacterial microbiome and mycobiome associated with PFR were demonstrated. However, in particular, no ocular bacterial or fungal pathogen was identified in the PFR group compared with the control group (13, 14). Viromes have been previously characterized on the surface of the skin (15), blood (16, 17), breast milk (18), cerebrospinal fluids (19), oral cavity (20, 21), lower gastrointestinal tract (22–24), respiratory tract (25), bladder (26), and vagina (27, 28). This is probably the first study on the virome of the vitreous fluid of the human eye of normal healthy individuals and is compared with that on the virome of the vitreous fluid of patients with PFR and retinitis. Such studies would highlight the virome associated with the vitreous fluid of normal healthy eye and further by comparison with the vitreous fluid of PFR and retinitis group patients, the data would enhance the understanding of the role of these viruses in PFR, an ocular disease.

## Materials and Methods

### Study Site

Virome in the vitreous fluid that was collected from study participants who were scheduled for pars plana vitrectomy/vitreous biopsy as part of their treatment was studied. Vitreous biopsy was performed in the operating room under full aseptic conditions by trained vitreoretinal surgeons. There was no difference in the preparation for PPV and vitreous biopsy.

### Collection of Vitreous Fluid From Control Individuals

Collection of vitreous fluid from healthy controls was not permitted because of ethical reasons. Therefore, in this cohort, we collected vitreous fluid from individuals who were to undergo ocular procedures for non-infectious ocular disorders such as a macular hole and rhegmatogenous detachment. The inclusion criteria include all individuals aged above 18 years without any systemic infection 3 months prior to the vitreous biopsy. The exclusion criteria for this cohort included individuals with uncontrolled glaucoma, diabetes and hypertension, and fever due to systemic infection. These controls were not symptomatic for post-fever retinitis (PFR) and had no other ocular infection. Vitreous fluid (300 μl) was collected from each individual in the control group (*n* = 16) through pars plana vitrectomy/vitreous biopsy by an ophthalmologist (Supplementary Table 1) and stored at −80°C until it was used. The study was designed and conducted according to the tenets of the Declaration of Helsinki.

### Collection of Vitreous Fluid From Individuals With Post-fever Retinitis or Other Retinitis

The PFR group included individuals with retinitis that normally manifests 2–4 weeks post-systemic febrile fever. The cause of the systemic fever in two individuals was typhoid, while in others the cause of fever was not identified (Supplementary Table 1). All individuals with PFR who had a history of inflammatory disorders of the eye, uncontrolled glaucoma, hypertension, and diabetes were excluded from the study. Vitreous fluid (300 μl) was collected from these individuals with PFR and retinitis (*n* = 9) by an ophthalmologist using the procedure described above (Supplementary Table 1) and stored at −80°C until it was used. This part of the study was also approved by the above ethics committee (Ethics reference number LEC 09-17-079 dated September 1, 2017 to August 31, 2019). The study was designed and conducted according to the tenets of the Declaration of Helsinki.

### Nucleic Acid Extraction and Metagenome Sequencing

Deoxyribonucleic acid/RNA was extracted from about 200 μl of the vitreous sample using a PureLink DNA/RNA extraction kit (Thermo Fisher Scientific, Mumbai, India) and according to the protocol of the manufacturer. The extracted DNA was quantified using a Qubit 3.0 fluorometer (Thermo Fisher Scientific, Carlsbad, CA, United States) and visualized by gel electrophoresis on a 1% (w/v) agarose gel. The extracted nucleic acids were amplified with random hexamers using an amplification kit (TransPlex, Sigma Aldrich Chemicals Private Limited, St. Louis, MO, United States). For library preparation and sequencing, NEBNext Ultra DNA Library Prep Kit for Illumina Nextseq 500 PE sequencing protocol was followed by paired-end sequencing with 2 bp × 150 bp chemistry on the Illumina Nextseq 500 platform. Care was taken to avoid microbial contamination from the environment by carrying out all the steps such as sample preparation, DNA extraction, PCR, and whole genome amplification procedures in a dedicated laminar flow hood. Sterile water was used as a negative control instead of template DNA in PCR, and, consistently, amplification was negative, implying a lack of contaminating DNA. No virus sequences could be generated from the negative controls.

### Viral Metagenomic Analysis

FASTQ files of the raw reads were generated for all the 25 samples that were sequenced. These raw sequence reads were analyzed for quality parameters, such as read length, phred quality score (<25), GC (guanine and cytosine) content, and presence of ambiguous bases. Sequencing adapters from the raw sequences were trimmed using trim-galore (version 0.4.0) (29) and Cutadapt version 1.2 (30). Subsequently, all the reads were subjected to the FastQC (version 0.11.3) tool, which helped in identifying reads with a quality score >Q25. Human genome sequences were removed using decontam (github.com/benjjneb/decontam) and Bowtie 2 tools using NCBI-GRCh38 as the reference genome. Contigs were analyzed for viral annotations using viral reference sequence IDs from NCBI (ftp://ftp.ncbi.nlm.nih.gov/refseq/release/viral/ - ftp://ftp.ncbi.nlm.nih.gov/refseq/release/viral/viral.3.protein.faa.gz). Refseq IDs of NCBI were converted to GI (geninfo) numbers and used for functional annotation. The resultant files (.aln) were opened in MEGAN 5. Post-viral annotation, Meta Genome Analyzer (MEGAN) v 5.11.3 was used for comparative analysis of the samples and generation of the virome biome file for the 25 samples. The biome data file comprising the abundance of all the viruses is provided in Supplementary Table 2. The MEGAN tool was also used for deriving KEGG pathway analysis.

### Statistical Analysis

The vegan package in R (http://vegan.r-forge.r-project.org/) was used to generate rarefaction curves and for quantifying diversity indices. The batch effect in the viromes was removed using the ComBat function in the package SVA (31). Alpha diversity indices viz., Shannon diversity, Simpson index, and the observed number of viral genera were calculated, and the degree of variation in the viromes of the groups was ascertained. An unpaired *t*-test was conducted using GraphPad Prism to determine the statistical significance of alpha diversity indices (https://www.graphpad.com/quickcalcs/ttest2/). Significant changes between VC and PFR were ascertained by Kruskal–Wallis and Wilcoxon signed rank tests (with *P* < 0.05 as significant). Similarly, the Kruskal–Wallis and Wilcoxon signed rank tests were conducted to determine the significant changes in the KEGG pathways between VC and PFR. In addition to these, a linear discriminant effect size (LEfSe) analysis was performed to identify discriminate viral genera (http://huttenhower.sph.harvard.edu/galaxy/). To visualize the relative abundances-based clustering of the genera, a rank-sum normalized heat map was generated for the viral genera of both cohorts. A principal coordinate analysis (PCoA) plot was generated for the 25 viromes using the ade4 package in R (v3.2.5) by employing Jensen–Shannon divergence distance metric *K*-means clustering (*k* = 2).

### STRING Network Analysis

Functional predictions for the significantly abundant KEGG pathways in the PFR group were ascertained by STRING (Search Tool for the Retrieval of Interacting Genes/Proteins) network analysis. For this, the KEGG orthology data were subjected to the KEGG Mapper tool to get the protein/gene IDs. These IDs were then used to ascertain the protein-protein associations in the STRING network analysis tool (version-11-0.string-db.org/) (32). K-means clustering was employed to visualize the cluster of proteins.

### Correlation Network Analysis of Viral Genera

Correlation Network (33) is a Cytoscape (34) plugin that was used to detect interactive networks of the discriminative viral genera in the VC and PFR groups independently. Spearman correlation coefficient (*r*) was used to analyze the interactions among the different discriminative viral genera (mutual exclusions/negative and co-presence/positive interactions).

## Results

### Metagenomes of the Vitreous Samples and the Virome

**Figure 1:**
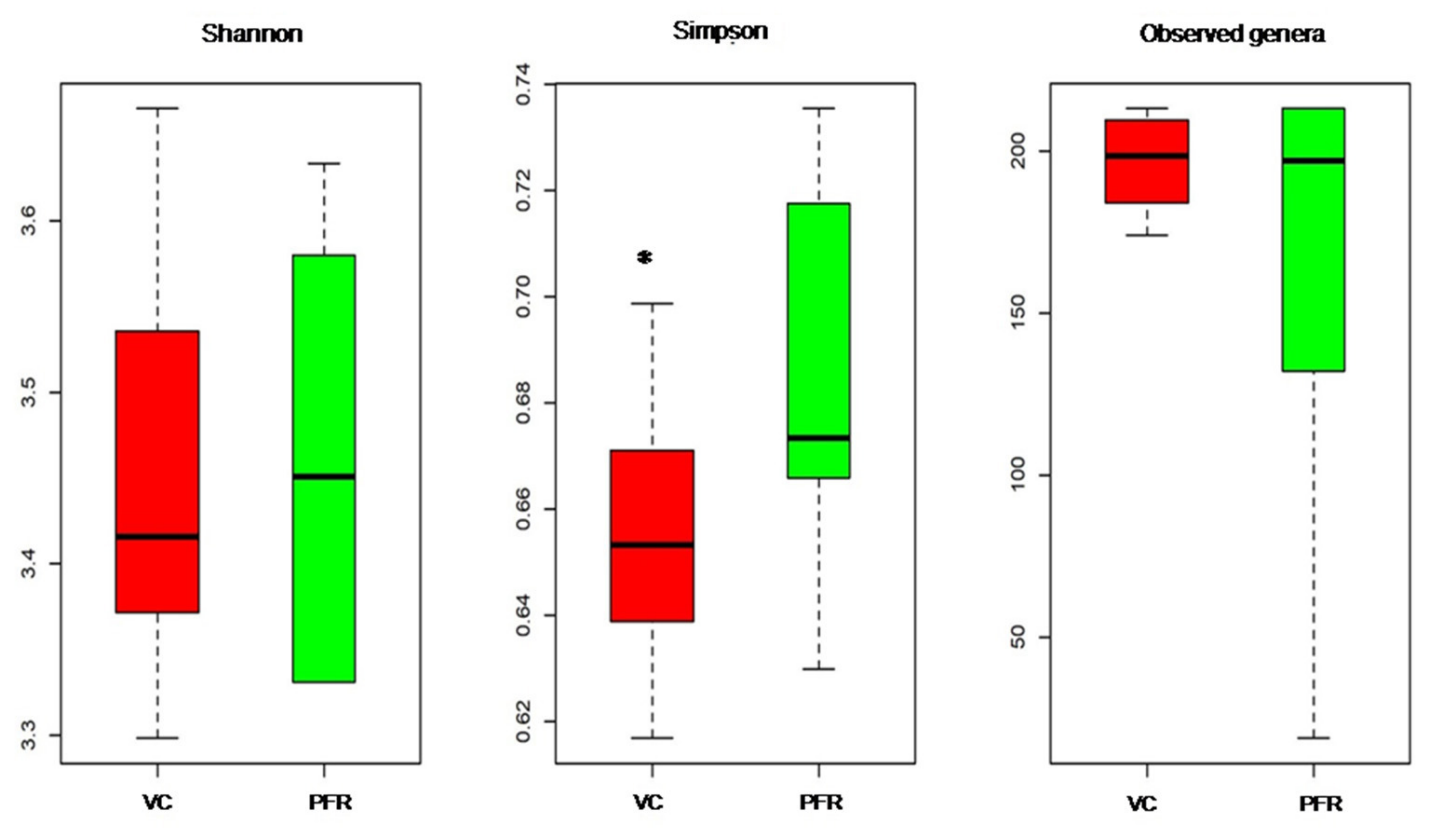
Box-plots depicting the Alpha diversity indices of the viromes in the vitreous fluid of controls (VC) and post-fever retinitis (PFR) groups.*Indicates a significant difference between VC and PFR (*p* < 0.05).

**Figure 2:**
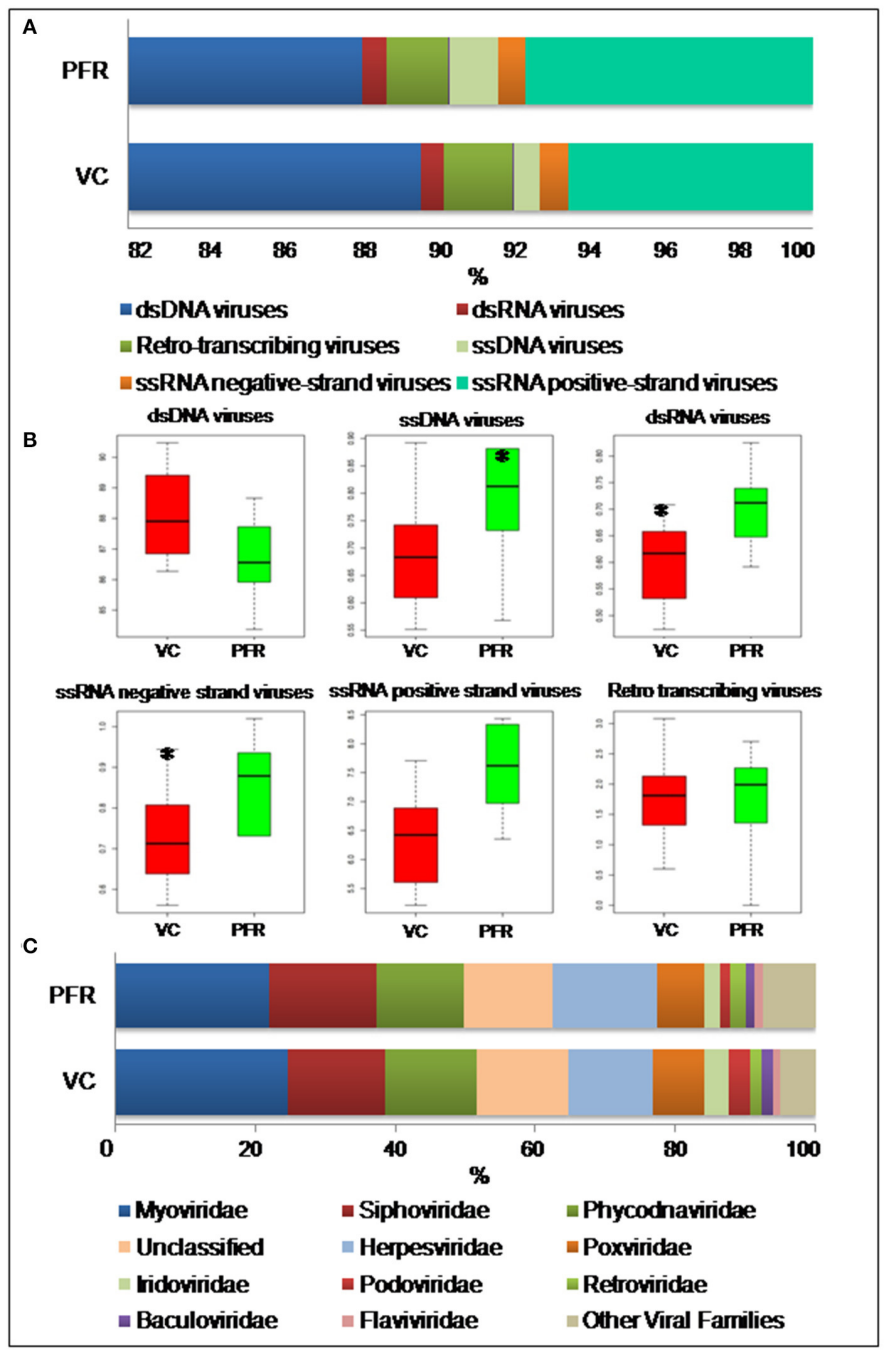
**(A)** Abundance of different virus groups in the vitreous fluid of control (VC) and post-fever retinitis (PFR) groups. **(B)** Box plots illustrating the abundance of dsDNA viruses, ssDNA viruses, dsRNA viruses, ssRNA negative-strand viruses, ssRNA positive-strand viruses, and Retro transcribing viruses in the vitreous fluid of control (VC) and post-fever retinitis (PFR) groups. **(C)** Abundance of Virus families in the vitreous fluid of control (VC) and post-fever retinitis (PFR) groups. *indicates significant change (*p* > 0.05).

A total of 96.3 million reads were generated for all the 25 vitreous samples of control (VC, *n* = 16), PFR (*n* = 6), and retinitis (RET) (*n* = 3) groups. In order to understand the differences and similarities among the VC, PFR, and RET samples, PCA, a statistical tool, was employed, and this analysis showed that all the three non-PFR and six PFR clustered together compared with the control (VC) samples (Supplementary Figure 1). Furthermore, since both PFR and RET manifest as retinitis, henceforth all the data of the two cohorts would be combined and designated as PFR and analyzed. Reads assigned to viruses comprised a total of 1,016,037 with 617,874 and 398,163 reads assigned to viruses in the control (VC) and PFR groups, respectively (Table 1). Rarefaction curves of the viral microbiomes (virome) showed a tendency toward saturation, indicating that majority of the viral diversity was identified at the genera level (Supplementary Figure 2). An alpha diversity analysis showed a significant difference in Simpson diversity indices between the VC and PFR groups; a significant difference was not detected in Shannon and the observed number of genera (Figure 1). Viral groups, such as double-stranded DNA (dsDNA) viruses, double-stranded RNA (dsRNA) viruses, retrotranscribing viruses, single-stranded DNA (ssDNA) viruses, single-stranded RNA (ssRNA) positive-strand viruses, and ssRNA negative-strand viruses were detected in both the VC and PFR groups (Figure 2A). ssDNA, dsRNA, and ssRNA viruses showed significant differences between the control and PFR groups (*P* < 0.05; Figure 2B). Except for dsDNA viruses, all the other viruses were increased in the PFR group compared with the VC group. dsDNA viruses are predominantly present in both groups with a mean abundance of 88.1 and 87.1 in the control and PFR groups, respectively (Supplementary Table 3). Among the RNA viruses, ssRNA positive-strand viruses are abundant, having a mean abundance of 6.3 and 7.5 in the control and PFR groups, respectively.

**Table 1:** Reads assigned to viruses in the vitreous fluid of control (VC) and post-fever retinitis (PFR) groups.

**Sample groups**		**Reads in millions (*Q* > 25)**	**Reads assigned to viruses**
VC	Total	48.9	617,874
	Average	3.88	38,617
PFR	Total	47.4	398,163
	Average	5.27	44,240
VC+PFR	Total	109.5	1,016,037
	Average	4.38	41,428

**Figure 3:**
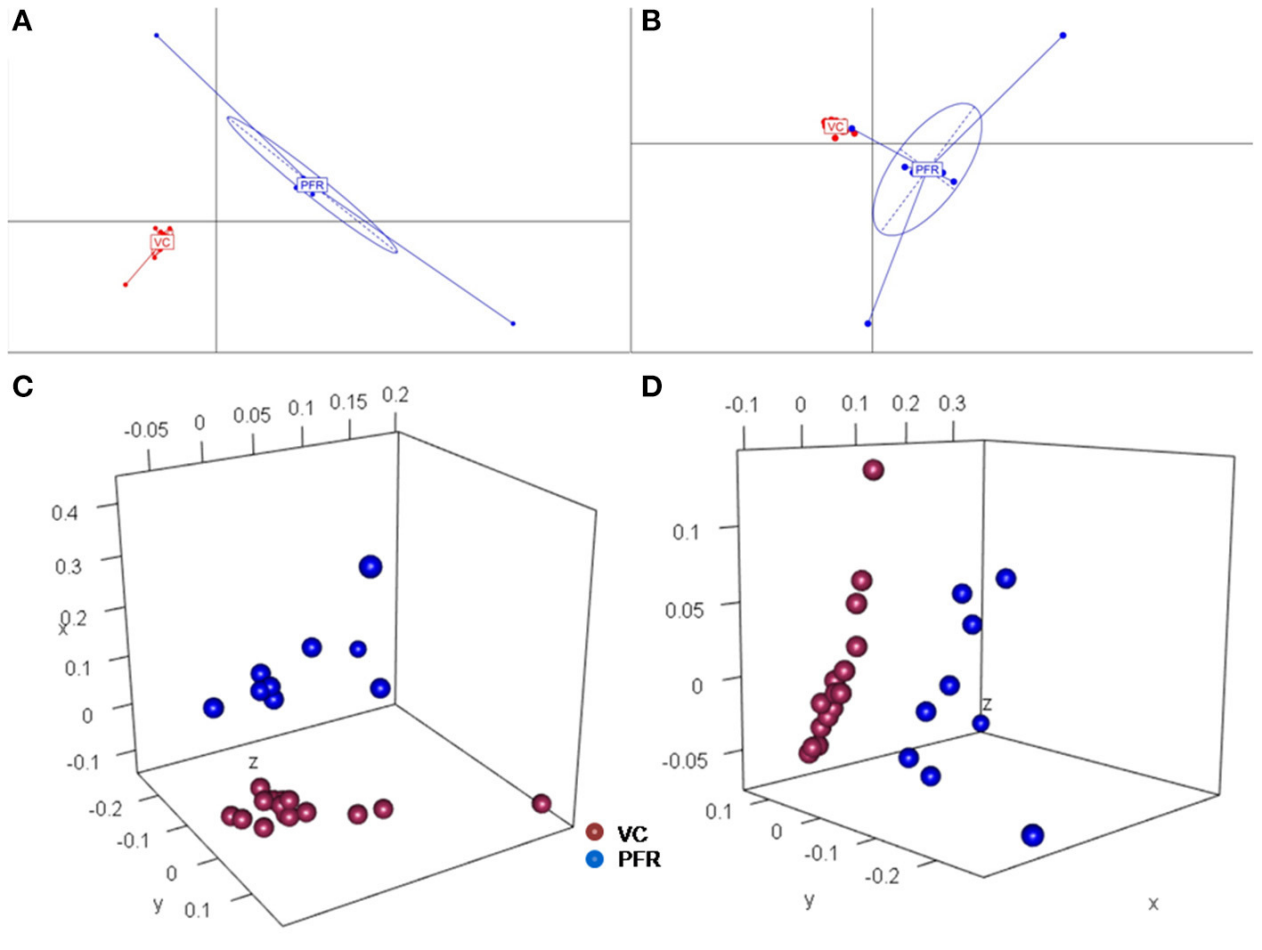
Principal coordinate (PCoA) and Non-metric multidimensional scaling (NMDS) analysis in controls (VC) and post-fever retinitis (PFR) groups. PCoA plots are based on Jacard distances of eukaryotic viral genera **(A)** and Bacteriophages **(B)**. Three-dimensional NMDS are based on Bray-Curtis distances of Eukaryotic viruses **(C)** and Bacteriophages **(D)**.

**Figure 4:**
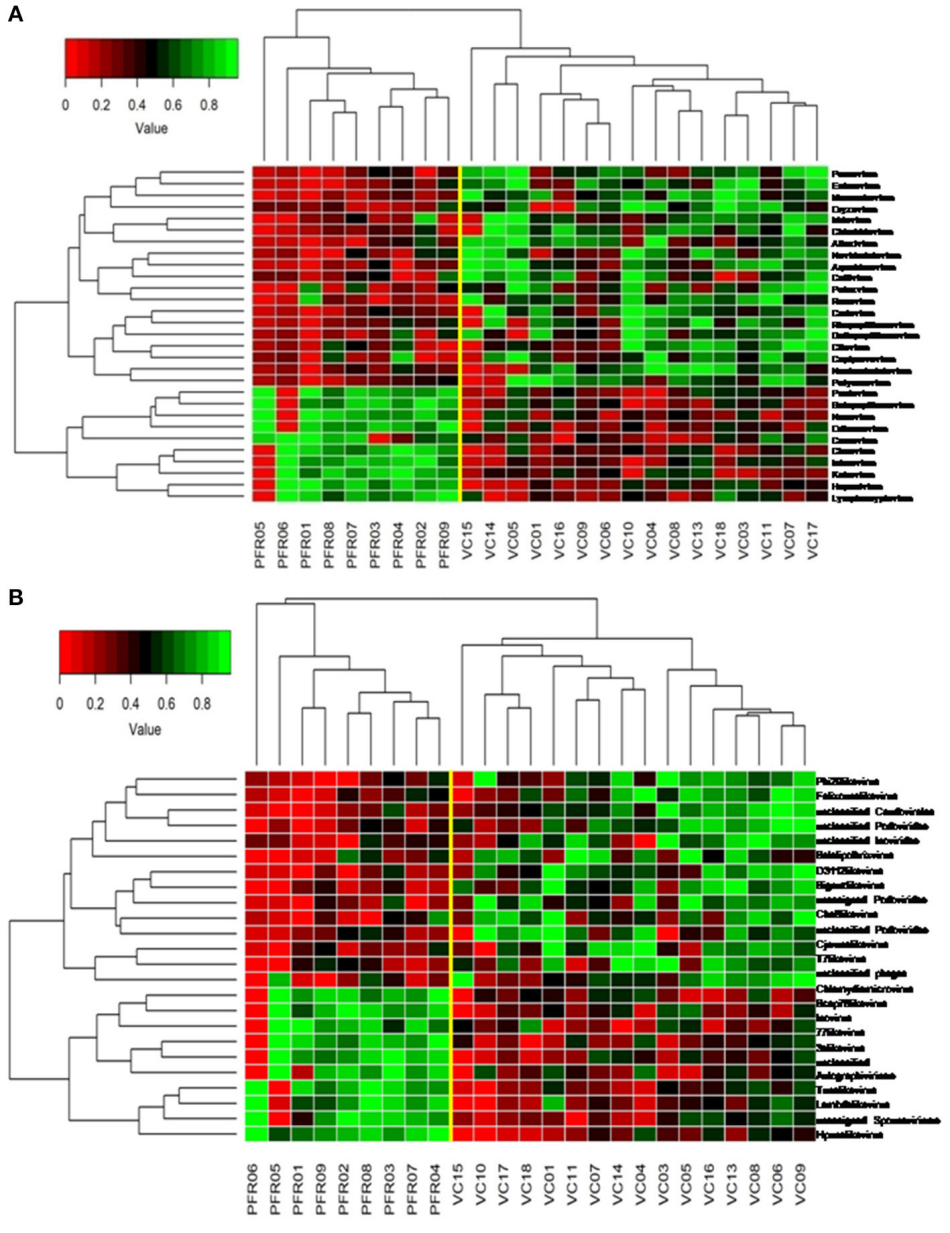
Two-dimensional heat map showing rank normalized abundances (scaled between 0 and 1) in the control group (VC) and post-fever retinitis (PFR) group of eukaryotic viruses **(A)** and bacteriophages **(B)**.

**Figure 5:**
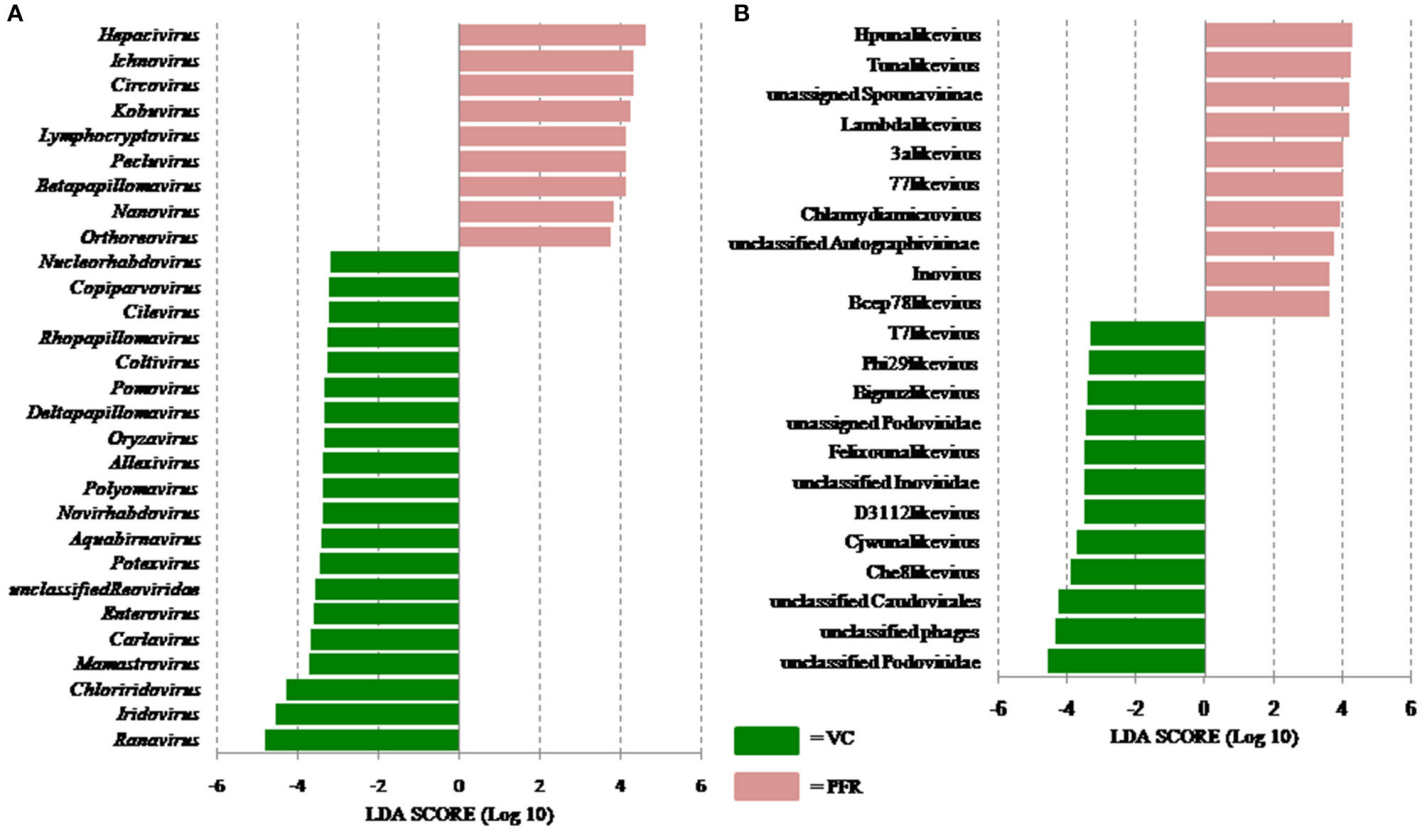
Linear discriminant analysis (LDA) of the virome of VC and PFR. **(A)** LDA in Eukaryotic viruses and **(B)** LDA in Bacteriophages. The bars in the figure represent the statistically significant genera as determined by the linear discriminant analysis (LDA) combined with effect size measurements (LEfSe). Peach color bars indicate an increase in the relative abundance of the genera in the PFR group.

A total of 68 viral families were identified in both the control and PFR groups (Supplementary Table 4). Three viral families, namely, *Astroviridae, Birnaviridae*, and *Polyomaviridae* were significantly different in the PFR group compared with the control (VC) group. Subsequently, at the genus level, 213 viruses were identified in all the 25 vitreous samples analyzed (Supplementary Table 2). Out of the 213 viruses, by non-parametric Kruskal-Wallis analysis, 54 genera, including nine unclassified or unassigned viruses, were significantly differentially abundant between the control (VC) and PFR groups. Out of the 54 discriminative genera, 30 were identified as eukaryotic viral genera (Table 2A) and 24 as bacteriophages (Table 2B). The principal coordinate analysis (PCoA) (Figures 3A–D) and the heat map analysis (Figures 4A,B) revealed a clear distinctive difference between the viromes of the control and PFR groups. The PCA was performed for the significantly differentially abundant eukaryotic viruses and bacteriophages between the control (VC) and PFR groups (Figures 3A,B). This analysis showed two distinct clusters for the VC and PFR groups. Simultaneously, 3 Dimensional Non-metric Multidimensional Scaling (NMDS) analysis also showed that the eukaryotic and bacteriophage viromes of the PFR group formed a distinct cluster from the control group (Figures 3C,D). Linear discriminant analysis (LDA), combined with effect size measurements (LEfSe) analysis, indicated a significant increase or decrease in the abundance of the 29 eukaryotic viral genera (Figure 5A) and 22 bacteriophages (Figure 5B).

**Table 2A:** Discriminative viral genera in the VC and PFR groups (*P* < 0.05).

**Sl No**.	**Viral genera**	**Mean abundance VC**	**Mean abundance PFR**	***P-*value (VC vs. PFR)**
1.	*Lymphocryptovirus*	0.488078	0.819065	0.008
2.	*Chloriridovirus*	0.737222	0.559702	0.008
3.	*Iridovirus*	2.013551	1.763227	0.021
4.	*Ranavirus*	2.177961	1.480304	0.004
5.	*Betapapillomavirus*	0.012454	0.187051	0.006
6.	*Deltapapillomavirus*	0.029408	0.017996	0.018
7.	*Rhopapillomavirus*	0.028845	0.018844	0.036
8.	*Ichnovirus*	0.010591	0.375047	0.006
9.	*Polyomavirus*	0.055518	0.03307	0.015
10.	*Aquabirnavirus*	0.040861	0.017954	0.001
11.	*Coltivirus*	0.017641	0.009325	0.032
12.	*Orthoreovirus*	0.048721	0.129131	0.006
13.	*Oryzavirus*	0.00604	0.001589	0.02
14.	*Circovirus*	0.025268	0.382411	0.007
15.	*Nanovirus*	0.013747	0.098435	0.006
16.	*Copiparvovirus*	0.018266	0.010248	0.039
17.	*Novirhabdovirus*	0.019379	0.010984	0.012
18.	*Nucleorhabdovirus*	0.030137	0.019842	0.036
19.	*Mamastrovirus*	0.096279	0.036622	0.001
20.	*Cilevirus*	0.015397	0.007587	0.043
21.	*Hepacivirus*	0.242584	0.981777	0.006
22.	*Enterovirus*	0.07924	0.042516	0.001
23.	*Kobuvirus*	0.089026	0.414933	0.006
24.	*Comovirus*	0.031522	0.064082	0.049
25.	*Allexivirus*	0.027912	0.016201	0.007
26.	*Potexvirus*	0.124534	0.103455	0.035
27.	*Carlavirus*	0.143193	0.096071	0.004
28.	*Pecluvirus*	0.011913	0.192004	0.006
29.	*Pomovirus*	0.031057	0.017243	0.004
30.	Unclassified *Reoviridae*	0.006582	0.002714	0.027

**Table 2B:** Discriminative bacteriophages in the VC and PFR groups (*P* < 0.05).

**Sl No**.	**Bacteriophage genera**	**Mean abundance VC**	**Mean abundance PFR**	***P-*value (VC vs. PFR)**
1.	*Bcep78likevirus*	0.067	0.139	0.008
2.	*Felixounalikevirus*	0.180	0.131	0.018
3.	*Hpunalikevirus*	0.118	0.496	0.001
4.	*T7likevirus*	0.167	0.134	0.015
5.	*Phi29likevirus*	0.042	0.022	0.006
6.	*3alikevirus*	0.148	0.362	0.006
7.	*77likevirus*	0.169	0.366	0.006
8.	*Bignuzlikevirus*	0.106	0.074	0.002
9.	*Che8likevirus*	0.696	0.534	0.025
10.	*Cjwunalikevirus*	0.467	0.356	0.02
11.	*D3112likevirus*	0.135	0.083	0.002
12.	*Lambdalikevirus*	0.445	0.715	0.011
13.	*Tunalikevirus*	0.112	0.440	0.006
14.	*Betalipothrixvirus*	0.405	0.341	0.037
15.	*Inovirus*	0.105	0.171	0.011
16.	*Chlamydiamicrovirus*	0.013	0.184	0.006
17.	Unassigned Spounavirinae	0.634	0.923	0.016
18.	Unclassified Autographivirinae	0.152	0.271	0.042
19.	Unclassified Picovirinae	0.096	0.073	0.036
20.	Unassigned Podoviridae	0.153	0.107	0.004
21.	Unclassified Podoviridae	2.075	1.351	0.005
22.	Unclassified Caudovirales	1.452	1.068	0.002
23.	Unclassified Inoviridae	0.009	0.004	0.045
24.	Unclassified phages	1.832	1.404	0.044

Out of the 29 discriminative eukaryotic viruses, human viruses that significantly increased in the PFR group included *Hepacivirus, Circovirus, Kobuvirus, Lymphocryptovirus* (Epstein-Barr virus), and *Betapapillomavirus* (Figure 5A). Among bacteriophages genera, such as *Hpunalikevirus* (Haemophilus phage HP1), *Tunalikevirus* (Enterobacteria phage T1), *Lambdalikevirus* (Escherichia virus Lambda), *77likevirus* (Staphylococcus phage 77), *Chlamydiamicrovirus* (Chalmydia phage 1), *Inovirus* (Escherichia virus M13), and *Bcep78likevirus* (Burcholderia phage) increased in the PFR group compared with the VC group (Figure 5B).

### Predominant Viruses in PFR Vitreous Samples

**Figure 6:**
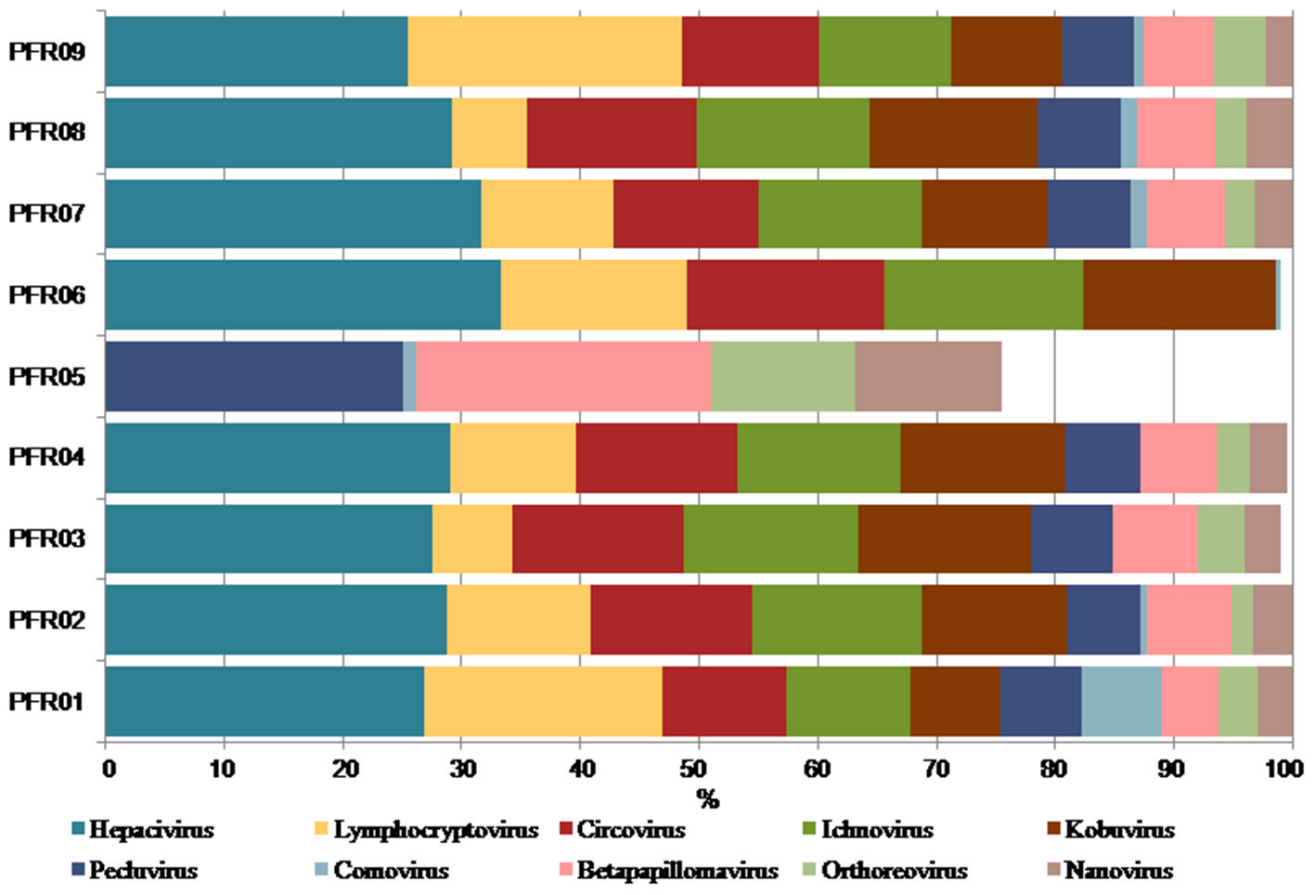
Increase in the abundance of discriminative viral genera in PFR samples compared to mean abundance in the control group.

The relative abundance of the discriminative human viruses in each of the PFR samples is visualized in the bar graph (Figure 6). A total of 10 viruses were consistently increased in the PFR samples compared with the VC samples. *Hepacivirus* was predominantly present in all patient samples of the PFR group except PFR05, and the abundance increase, compared with the control, ranged from 0 in PFR05 to 40.35% in PFR06. Furthermore, in all the samples, excluding PFR05, an ocular pathogenic virus namely Lymphocryptovirus (Epstein-Barr Virus) was identified.

### KEGG Pathway Analysis

**Figure 7:**
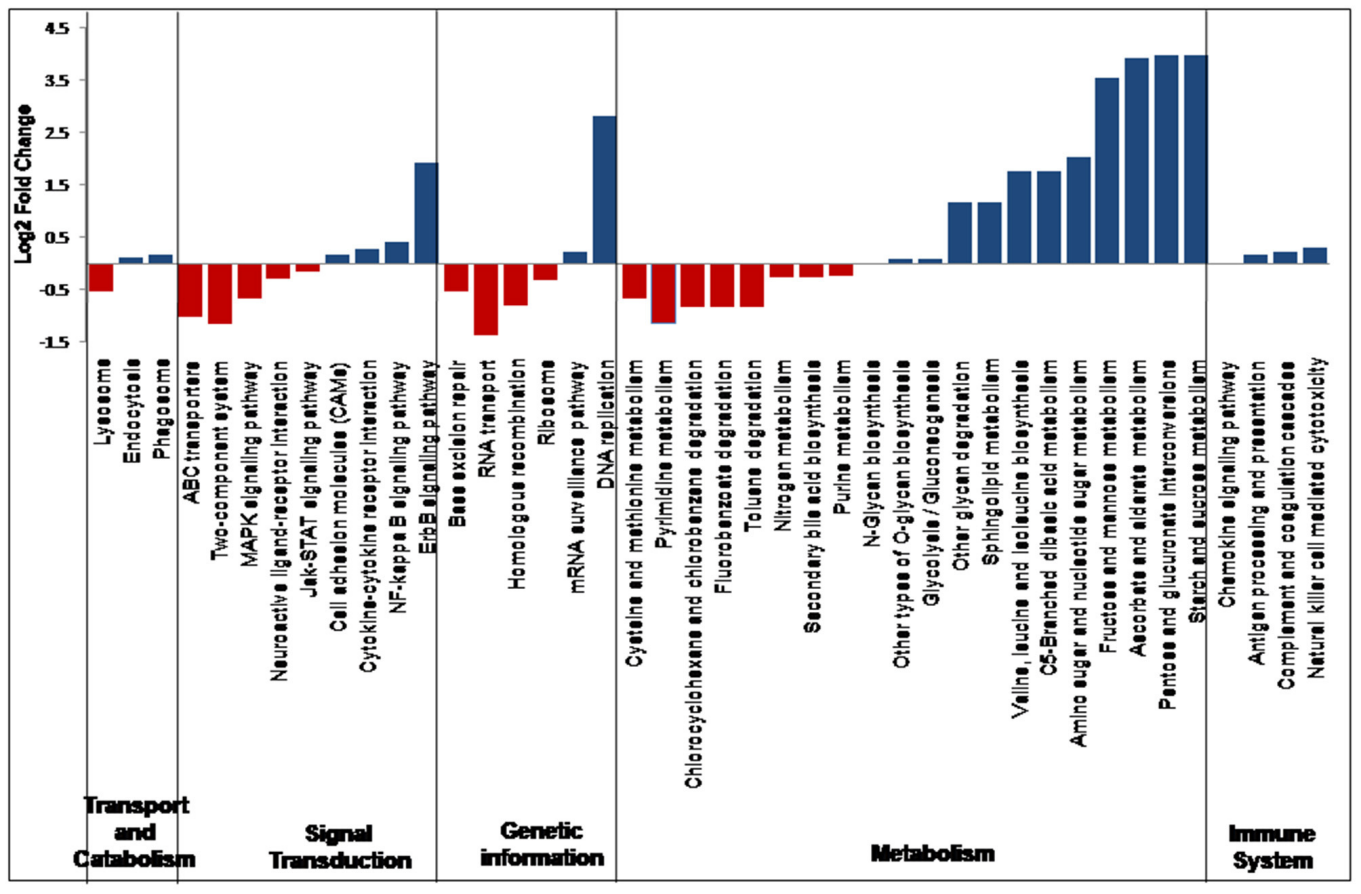
Differential abundance in the discriminative KEGG pathways in VC compared to PFR groups.

In all the 25 samples, pathways belonging to transport and catabolism, signal transduction, metabolism, immune system, and other pathways were identified. The Kruskal–Wallis statistical analysis identified 41 pathways as significantly different between the VC and PFR groups (Supplementary Table 5; Figure 7). Out of the three pathways belonging to transport and catabolism, pathways such as endocytosis and phagosome increased in the PFR group compared with the VC group. In addition, several signaling transduction pathways, such as the ErbB signaling pathway, NF-kappa B signaling pathway, signaling molecules and interaction, cytokine-cytokine receptor interaction, major histocompatibility complex, and class I, were increased in PFR compared with control. Several metabolic pathways, such as glycolysis/glucogenesis, tropane, piperidine and pyridine alkaloid biosynthesis, glycan biosynthesis, pyruvate, amino acid and nucleotide sugar metabolism; sphingolipid, glycine, serine, and threonine metabolism; valine, leucine, and isoleucine biosynthesis, and pyrimidine metabolic pathways were also significantly increased in the PFR group compared with the control group. All the pathways of the immune system, such as chemokine signaling pathway, antigen processing and presentation, complement and coagulase cascades, hematopoietic cell lineage, and natural killer cell-mediated cytotoxicity, were significantly increased in PFR compared with the control group. The STRING network analysis of the KEGG orthologs depicted pathways belonging to Epstein-Barr virus infection, influenza A pathway, cytokine-cytokine receptor interaction, Jak-STAT signaling pathway, and complement and coagulation cascades (Supplementary Figure 3).

## Discussion

Viral tropism toward immune privileged sites within the body, such as eyes, was demonstrated in animal model studies (35, 36), but its role in health and disease was not understood. Viruses such as *Herpesvirus* and *Flaviviruses* may establish latent infection in neuronal cells and epithelial cells, respectively (37, 38). It is also becoming more obvious with reports on the virome from different body fluids, such as cerebrospinal fluids (CSF), human milk, and blood that viruses could reach those parts of the body that were once thought to be sterile (19). In this study, we reported the virome in the vitreous fluid of healthy individuals compared with that of individuals with retinitis. Significant changes in the viral diversity were observed between the control and patient groups (Figure 1). In the vitreous fluids of healthy and patient samples, sequences of viruses having both DNA and RNA as their genetic material were identified. Based on host specificity viruses like bacteriophages and eukaryotic viruses comprising human, animal, and plant viruses were identified (Tables 2A,B; Figures 2A–C). Earlier virome studies on human body fluids also reported similar results (19). A comparison of viral families of vitreous fluids with other studies comprising body fluids, such as cerebrospinal fluid and plasma, reveal that families such as *Myoviridae, Siphoviridae, Phycodnaviridae, Podoviridae, Herpesviridae, Podoviridae, Microviridae, Inoviridae, Poxviridae*, and *Papilliomaviridae* were common (19). The top 10 abundant viral families in the vitreous fluid of all the 25 samples include *Myoviridae, Siphoviridae, Herpesviridiae, Phycodnaviridae, Poxviridae, Iridoviridae, Podoviridae, Baculoviridae, Flaviviridae*, and *Retroviridae* (Figure 2C). Furthermore, viruses *Myoviridiae, Siphoviridae*, and *Phycodnaviridae* were also reported as the largest families in the CSF and plasma (Supplementary Table 4; Figure 2C) (19). A recent study on the ocular surface microbiome in children also revealed the presence of several viruses representing Eukaryotic viruses and bacteriophages (39). At the genera level, 30 Eukaryotic viral genera were significantly different between the control (VC) and post-fever retinitis (PFR) groups (Tables 2A,B), and a clear distinction between the groups was visualized by principal coordinate analysis (PCoA) and heat map analysis (Figures 3A–D, 4A,B). Ten viral genera showed a significant increase in abundance in the PRF group compared to the control group (Table 2; Figure 5A). Out of these 10 genera, surprisingly, only 1 viral genus, *Lymphocryptovirus*, that comprises the human pathogen Epstein-Barr virus was reported in ocular infection (40). A number of ocular diseases, such as oculoglandular syndrome, dry eye syndrome, dacryoadenitis, conjunctivitis, episcleritis, keratitis, uveitis, choroiditis, retinitis, retinal vasculitis, and papillitis, are associated with Epstein-Barr virus (EBV) infection (41). Therefore, the presence of *Lymphocryptovirus* in greater abundance in eight of the 9 PFR samples compared with the control would imply that this genus of the virus is predominant under pathological conditions (Figures 5, 6). The other discriminative genera that were increased in the PFR group include viruses such as *Hepacivirus, Betapappillomavirus, Orthoreovirus, Kobuvirus*, and *Circovirus* (Figures 5, 6). Several virome studies indicated the presence of *Papillomaviruses* in different parts of the body (25, 42, 43). The association between EBV and *Papillomavirus* was reported earlier with the epidemic of head and neck squamous cell carcinomas (HNSCC) (44), in co-infection and oral carcinogenesis (44), in patients with laryngeal, oropharyngeal, and oral cavity cancer (45), and in patients with type 2 diabetes mellitus (46). At the same time, the interaction of other viral genera, such as *Hepacivirus, Orthoreovirus, Kobuvirus*, and *Circovirus*, with EBV is not available in the literature.

Attempts have also been made to predict the coexistence and interactions among the viruses in healthy controls and patients with PFR by CoNet network analysis based on the abundance in the healthy and diseased state. This analysis helped in predicting the interactions of the ocular virus *Lymphocryptovirus* with other human viruses, such as *Hepacivirus, Circovirus*, and *Kobuvirus*, in the VC and PFR groups (Supplementary Figures 4A,B). In the VC group, *Hepacivirus* had a positive interaction, while *Circovirus* and *Kobuvirus* had a negative interaction with genus *Lymphocryptovirus*. However, in the PFR group both *Hepacivirus* and *Circovirus* had a positive interaction with genus *Lymphocryptovirus* and had no interaction with genus *Kobuvirus*. Whether such variations in interactions influence pathogenesis is hard to predict. Earlier, viral metagenomic studies have revealed the presence of single-stranded DNA viruses, such as *Anelloviruses* and *Circoviruses*, in the human body (47). The association of *Anelloviruses* with disease conditions was reported earlier in ocular fluids (48). In this study, although we could find *Anelloviruses* in the vitreous fluids of the patient group, their abundance was not significantly different from the healthy group. Alternatively, we observed a significant increase in *Circoviruses* that are taxonomically closely related to *Anelloviruses* in the PFR group (49).

Virus entry into a cell would mediate changes in pathways such as cellular processes, signaling molecules, transport and trafficking, and genome uncoating (50). In this study, significant changes in several pathways related to viral manifestation in the vitreous fluids were observed between the control and PFR groups (Figure 7). Increase in the pathways involving endocytosis and phagosomes in the patient group compared with the control group indicates active immune surveillance and the presence of pathogens (50, 51). Both the epidermal growth factor receptor (ErbB) and nuclear factor kappa B (NF-kB) signaling pathways were enhanced in the patient group, while the mitogen-activated protein kinase (MAPK) signaling pathway was decreased. This is in accordance with earlier studies that had indicated that ErbB family members are largely associated with infections because of different pathogens (52). It is also known that ErbB signaling pathways in turn would interact with mitogen-activated protein kinase (MAPK) and nuclear factor kappa B (NF-?B), etc. (52). The increased levels of cell adhesion molecules and cytokine-cytokine interaction pathways in the patient samples could also be due to active infection of EBV as indicated in the endothelial cells that were infected with EBV (53). Viral manifestation has been associated with the reprogramming of several host metabolic processes, which may include an increase in glycolysis, metabolic activity supporting the generation of nucleotides, amino acid generation, and lipid synthesis (54) (Figure 7). As a result of these changes in the host cells, several immune system-associated pathways were also increased in the patient group.

The STRING network analysis showed enrichment of KEGG pathways for the presence of Epstein-Barr infection and pathways belonging to the immune system (Supplementary Figure 2), thus suggesting the involvement of EBV in patients suffering from post-fever retinitis. Epstein-Barr Virus (EBV) occurs typically as an asymptomatic or paucisymptomatic infection during childhood and infects more than 95% of people worldwide (55). Thus, EBV may sustain as a latent virus in the host after primary infection (56). The results of this study do not suggest whether the EBV infection is primary or latent. Furthermore, presuming the prevalence of EBV in the majority of the population, it appears that the switch from latent to lytic could have had an important play in the individuals with PFR compared with the control group. On the other hand, several studies also indicated ophthalmic manifestation due to EBV infection in oculoglandular syndrome, dry eye syndrome, dacryoadenitis, conjunctivitis, episcleritis, keratitis, uveitis, choroiditis, retinitis, retinal vasculitis, and papillitis (57). At this juncture, it is worthwhile mentioning that the development of retinitis could have multiple etiological agents (bacteria, fungi, and viruses). The concomitant abundance of bacteriophages was significantly altered in the PFR group samples compared with the control group. Furthermore, we looked in the literature for the likely association of the EBV and discriminative bacterial genera, i.e., *Tannerella* and *Pimelobacter*, that was reported in our earlier publication in the PFR cohort (13). The direct association between *Tannerella* and *Pimelobacter* and EBV was not reported. However, in a periodontal disease study, significantly higher detection rates for genus *Tannerella* and EBV in a periodontitis group were observed (58). Nevertheless, the co-infection of EBV and fungi was reported occasionally. Heavy growth of *Aspergillus fumigatus* was found in the sputum along with Epstein-Barr virus (EBV) IgM in a patient who presented with a glandular fever-like illness and neutropenia (59). Similarly, a case of acquired hemophagocytic lymphohistiocytosis caused by dual infections with *Candida albicans* and reactivated EBV infections was reported recently (60). The nine pathogenic fungal genera that were significantly increased in PFR, *Setosphaeria, Arthroderma, Clavispora, Exserohilum, Paracoccidiodes, Pseudogymnoascus, Trichoderma, Kluveromyces*, and *Microsporum* were not reported to co-occur along with EBV infection (14). Therefore, further studies may be necessary to understand the likely involvement of bacterial and fungal genera along with EBV infection in PFR.

The metagenomic approach has allowed the identification of viruses in all patient samples that were otherwise not possible by routine molecular techniques. The major limitation of the study is that the results are based on a small number of participants with PFR. The number of participants remained low because of the rarity of the disease and ethical compliance. However, the study modestly justifies the findings and shows that there is an increase in the abundance of ocular pathogenic viruses in the majority of the patient samples. The results of the study imply that viral pathogens may co-exist in balance with the host in immunocompetent individuals and that this virus-host imbalance could have triggered the immune response in individuals with PFR.

## Data Availability Statement

The datasets presented in this study can be found in online repositories. The names of the repository/repositories and accession number(s) can be found in the article/Supplementary Material.

## Ethics Statement

The studies involving human participants were reviewed and approved by Institutional Review Board, L. V. Prasad Eye Institute, Hyderabad. The patients/participants provided their written informed consent to participate in this study.

## Author Contributions

KA researched data and wrote the manuscript. GS contributed to data curation and analysis. MT and RP collected the samples. SS contributed to the discussion and edited the manuscript. All authors contributed to the article and approved the submitted version.

## Conflict of Interest

The authors declare that the research was conducted in the absence of any commercial or financial relationships that could be construed as a potential conflict of interest.

## Publisher's Note

All claims expressed in this article are solely those of the authors and do not necessarily represent those of their affiliated organizations, or those of the publisher, the editors and the reviewers. Any product that may be evaluated in this article, or claim that may be made by its manufacturer, is not guaranteed or endorsed by the publisher.

## Supplementary Material

Supplementary Figure 1 Non-metric multidimensional scaling (NMDS) analysis of the controls (VC), post-fever retinitis (PFR), and non-post fever retinitis (RET) groups. Three-dimensional NMDS are based on Bray-Curtis distances of **(A)** eukaryotic viruses and **(B)** bacteriophages.

Supplementary Figure 2 Rarefaction curves of the viromes generated for the 25 vitreous samples collected from healthy controls (VC) and individuals with PFR.

Supplementary Figure 3 STRING network analysis depicting Kyoto Encyclopedia of Genes and Genomes (KEGG) pathways. The colors of the nodes indicate the interaction of the proteins in specific pathways. Edges indicate co-expression evidence. The thickness of the edge indicates the strength of available data support. Inter-cluster edges are represented as dashed lines.

Supplementary Figure 4 Correlation Network (CoNet) analysis of discriminative viral genera in **(A)** VC group and **(B)** PFR group. Nodes represent the genera, red color edges indicate negative interaction, and green color edges indicate positive interaction.

Supplementary Table 1 Demographic file comprising the details of control samples (VC, *n* = 16) and post-fever retinitis samples (PFR, *n* = 9).

Supplementary Table 2 Virome Biome file of control (VC) and post-fever retinitis (PFR) samples.

Supplementary Table 3 Relative abundance of different viral groups in the vitreous of Control (VC, *n* = 15) and post-fever retinitis (PFR, *n* = 9) groups.

Supplementary Table 4 Relative abundance of Viral families in the vitreous of Control (VC, *n* = 16) and post-fever retinitis (PFR, *n* = 9) groups.

Supplementary Table 5 KEGG pathways in VC and PFR groups (*p* < 0.05).
