# Supplementary figures and images for: Intraocular Viral Communities Associated With Post-fever Retinitis
Source: Front Med (Lausanne). 2021 Nov 19;8:724195. doi: 10.3389/fmed.2021.724195 (PMC8639604; doi:10.3389/fmed.2021.724195)

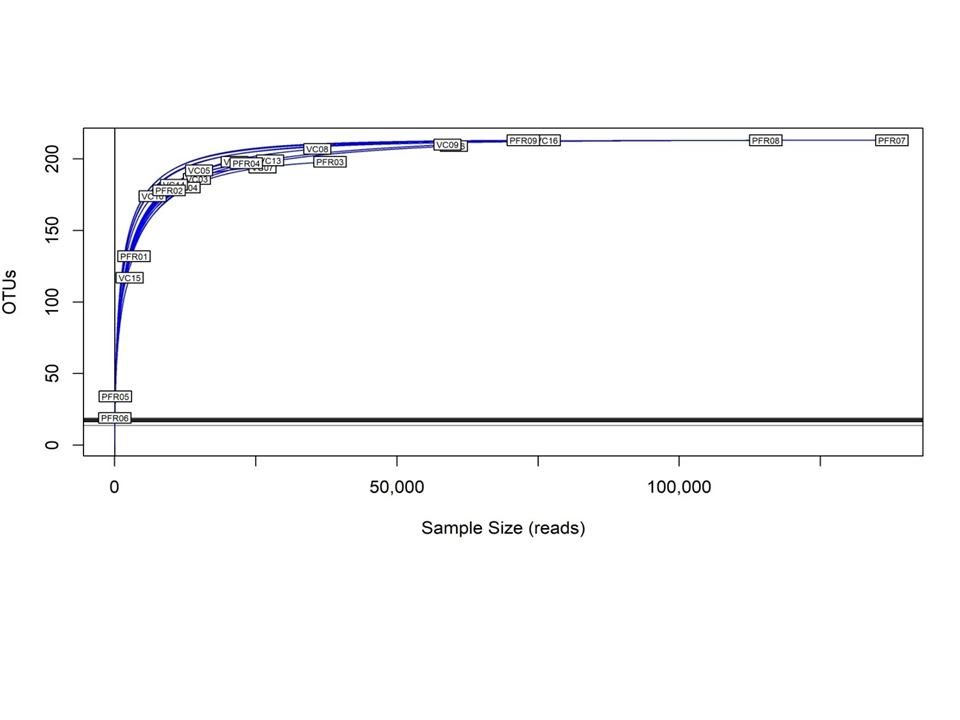

Supplement: Supplementary Figure 1 — Non-metric multidimensional scaling (NMDS) analysis of the controls (VC), post-fever retinitis (PFR), and non-post fever retinitis (RET) groups. Three-dimensional NMDS are based on Bray-Curtis distances of (A) eukaryotic viruses and (B) bacteriophages. [file Image_1.TIF]

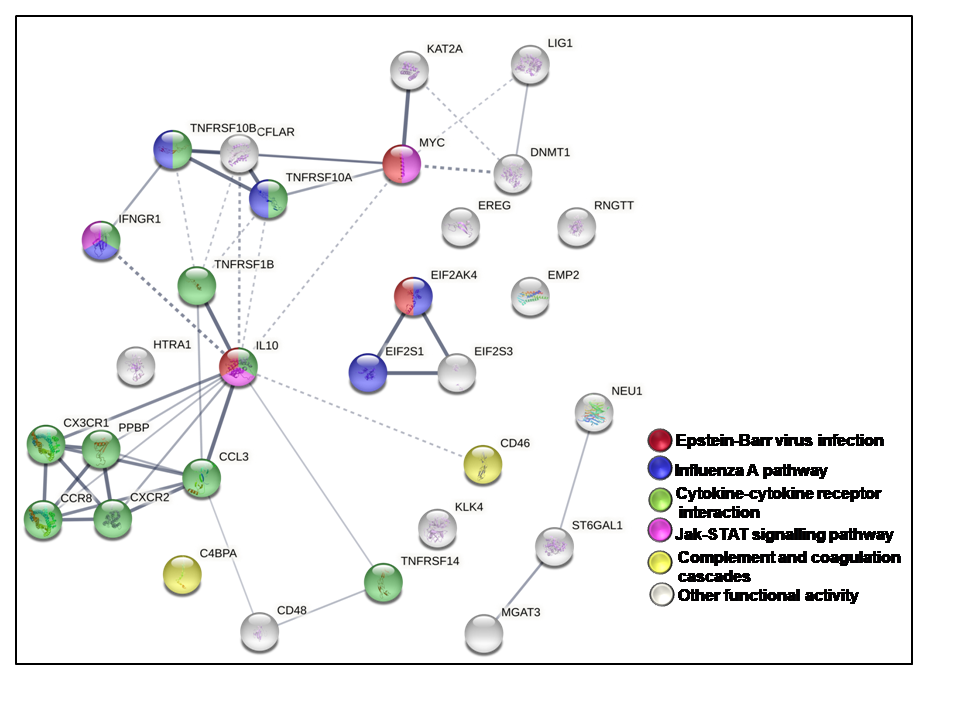

Supplement: Supplementary Figure 2 — Rarefaction curves of the viromes generated for the 25 vitreous samples collected from healthy controls (VC) and individuals with PFR. [file Image_2.TIF]

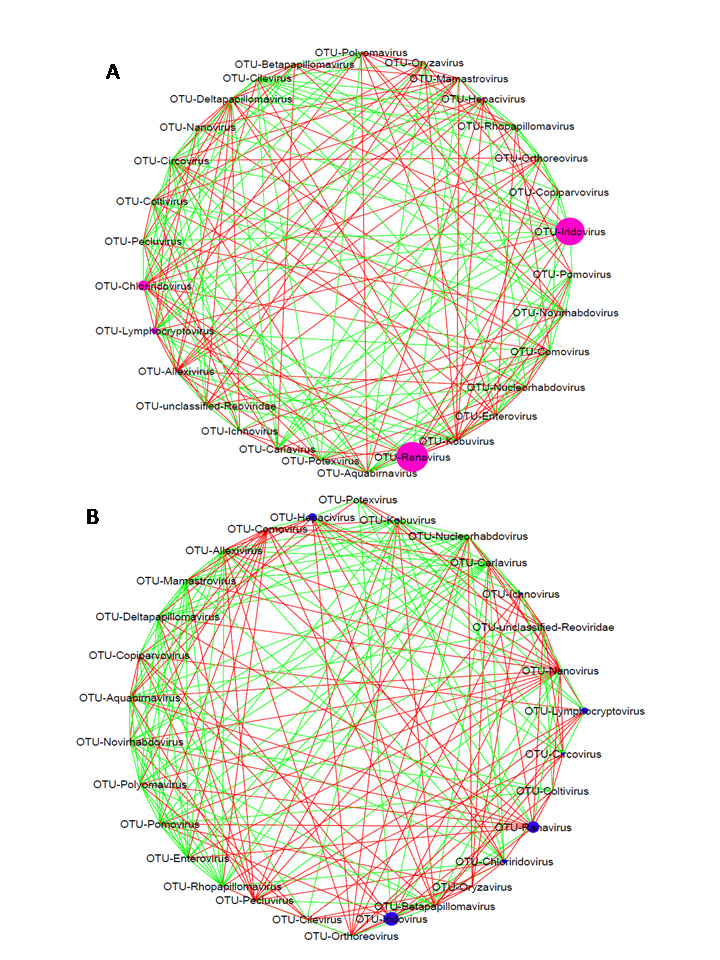

Supplement: Supplementary Figure 3 — STRING network analysis depicting Kyoto Encyclopedia of Genes and Genomes (KEGG) pathways. The colors of the nodes indicate the interaction of the proteins in specific pathways. Edges indicate co-expression evidence. The thickness of the edge indicates the strength of available data support. Inter-cluster edges are represented as dashed lines. [file Image_3.TIF]

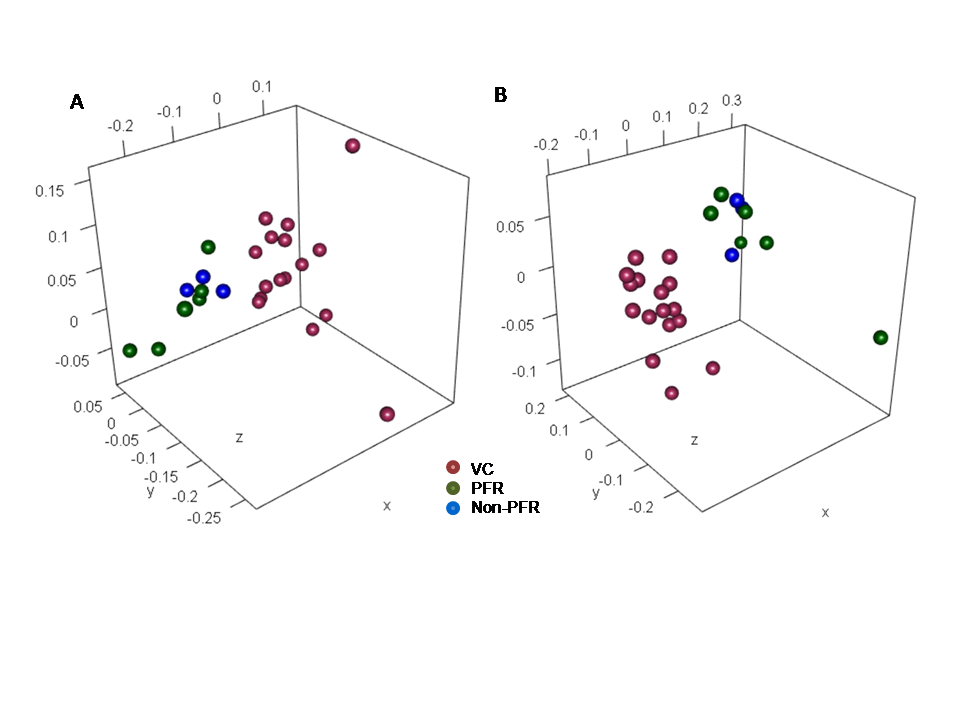

Supplement: Supplementary Figure 4 — Correlation Network (CoNet) analysis of discriminative viral genera in (A) VC group and (B) PFR group. Nodes represent the genera, red color edges indicate negative interaction, and green color edges indicate positive interaction. [file Image_4.TIF]
