# Supplementary material for: Intraocular Viral Communities Associated With Post-fever Retinitis
Source: Front Med (Lausanne). 2021 Nov 19;8:724195. doi: 10.3389/fmed.2021.724195 (PMC8639604; doi:10.3389/fmed.2021.724195)
Supplement: Supplementary Table 1 — Demographic file comprising the details of control samples (VC, n = 16) and post-fever retinitis samples (PFR, n = 9). [file Data_Sheet_1.docx]

**Supplementary Tables**

Table S1.Demographic file comprising the details of control samples (VC, n=16) and post fever retinitis samples (PFR, n=9)

| **Sl. No.** | **Sample ID** | **Age  (years)** | **Gender** | **State** | **Vitreous sample collection procedure** | **History of Retinitis** |
| --- | --- | --- | --- | --- | --- | --- |
|  | VC01 | 61 | Female | Telangana | Pars plana vitrectomy | nil |
|  | VC03 | 63 | Male | Madhya Pradesh | Pars plana vitrectomy | nil |
|  | VC04 | 47 | Male | West Bengal | Pars plana vitrectomy | nil |
|  | VC05 | 36 | Male | Andhra Pradesh | Pars plana vitrectomy | nil |
|  | VC06 | 36 | Male | Andhra Pradesh | Pars plana vitrectomy | nil |
|  | VC07 | 22 | Male | West Bengal | Pars plana vitrectomy | nil |
|  | VC08 | 57 | Male | Telangana | Pars plana vitrectomy | nil |
|  | VC09 | 67 | Female | Andhra Pradesh | Pars plana vitrectomy | nil |
|  | VC10 | 69 | Male | Maharashtra | Pars plana vitrectomy | nil |
|  | VC11 | 54 | Male | Telangana | Pars plana vitrectomy | nil |
|  | VC13 | 37 | Male | Telangana | Pars plana vitrectomy | nil |
|  | VC14 | 33 | Female | Andhra Pradesh | Pars plana vitrectomy | nil |
|  | VC15 | 68 | Female | Telangana | Pars plana vitrectomy | nil |
|  | VC16 | 42 | Male | Telangana | Pars plana vitrectomy | nil |
|  | VC17 | 44 | Male | Andhra Pradesh | Pars plana vitrectomy | nil |
|  | VC18 | 44 | Male | Andhra Pradesh | Pars plana vitrectomy | nil |
|  | PFR01 | 35 | Female | Maharashtra | Pars plana vitrectomy | Post febrile illness |
|  | PFR02 | 32 | Male | Telangana | Vitreous biopsy | Post Typhoid fever |
|  | PFR03 | 20 | Female | Karnataka | Vitreous biopsy | Post febrile illness |
|  | PFR04 | 42 | Male | Telangana | Vitreous biopsy | Post febrile illness |
|  | PFR05 | 39 | Male | Telangana | Vitreous biopsy | Post Typhoid fever |
|  | PFR06 | 40 | Female | Andhra Pradesh | Vitreous biopsy | Post febrile illness |
|  | PFR07 | 46 | Male | Telangana | Pars plana vitrectomy | Taxoplasma retinitis |
|  | PFR08 | 26 | Male | Telangana | Pars plana vitrectomy | Ocular tuberculosis |
|  | PFR09 | 46 | Male | Telangana | Vitreous biopsy | Viral retinitis |

Table S3. Relative abundance of different viral groups in the vitreous of Control (VC, n=15) and post fever retinitis (PFR, n=9) groups.

| Sl. No | Virus groups | Abundance in VC | | Abundance in PFR | |
| --- | --- | --- | --- | --- | --- |
|  |  | Mean | Range | Mean | Range |
| 1 | dsDNA viruses | 88.1 | 86.26- 90.47 | 87.1 | 84.37-91.67 |
| 2 | dsRNA viruses | 0.6 | 0.48-0.69 | 0.633 | 0.0-0.82 |
| 3 | Retro-transcribing viruses | 1.8 | 0.6- 3.01 | 1.6 | 0-0.2.7 |
| 4 | Satellites | 0.01 | 0.0-0.021 | 0.01 | 0.0- 0.02 |
| 5 | ssDNA viruses | 0.68 | 0.55-0.89 | 1.28 | 0.57-3.12 |
| 6 | ssRNA negative-strand viruses | 0.72 | 0.56-0.94 | 0.69 | 0.0-1.0 |
| 7 | ssRNA positive-strand viruses | 6.3 | 5.2-7.7 | 7.5 | 2.27-12.5 |

Table S4. Relative abundance of Viral families in the vitreous of Control (VC, n=16) and post fever retinitis (PFR, n=9) groups.

| Sl.No | Family | Abundance in Control group | | Abundance in PFR group | | p_value |
| --- | --- | --- | --- | --- | --- | --- |
|  |  | Range | Mean | Range | Mean |  |
|  | Astroviridae | 0.0355-0.0988 | 0.091101 | 0.0-0.073 | 0.037446 | 0.022 |
|  | Birnaviridae | 0.0334-0.1118 | 0.04789 | 0.0-0.0423 | 0.027486 | 0.039 |
|  | Polyomaviridae | 0.0-0.0763 | 0.055957 | 0.0-0.07 | 0.033824 | 0.047 |
|  | Betaflexiviridae | 0.0372-0.2096 | 0.163128 | 0.0-0.182 | 0.112461 | 0.095 |
|  | Podoviridae | 1.9404-3.7361 | 2.985588 | 0.0-3.479 | 2.274906 | 0.095 |
|  | Adenoviridae | 0.1953-0.3550 | 0.260978 | 0.0-0.381 | 0.233588 | 0.799 |
|  | Alphaflexiviridae | 0.1441-0.25 | 0.18205 | 0.0-0.247 | 0.151366 | 0.63 |
|  | Alphatetraviridae | 0.0-0.0148 | 0.006059 | 0.0-0.0293 | 0.005142 | 0.38 |
|  | Alvernaviridae | 0.0-0.02 | 0.007881 | 0.0-0.0293 | 0.008673 | 0.977 |
|  | Anelloviridae | 0.0078-0.0745 | 0.04008 | 0.0-0.073 | 0.035744 | 0.887 |
|  | Arenaviridae | 0.0445-0.174 | 0.113517 | 0.0-0.188 | 0.105244 | 0.932 |
|  | Arteriviridae | 0.0468-0.22 | 0.120043 | 0.0-0.294 | 0.136446 | 0.843 |
|  | Baculoviridae | 1.948-2.982 | 2.458687 | 0.0-3.125 | 2.295495 | 0.803 |
|  | Benyviridae | 0.0-0.01 | 0.005968 | 0.0-0.011 | 0.004055 | 0.622 |
|  | Bicaudaviridae | 0.171-0.2981 | 0.235379 | 0.0-0.757 | 0.292231 | 0.152 |
|  | Bromoviridae | 0.0-0.0274 | 0.018282 | 0.0-0.031 | 0.014176 | 0.61 |
|  | Bunyaviridae | 0.096-0.2189 | 0.131149 | 0.0-0.21 | 0.113262 | 0.671 |
|  | Caliciviridae | 0.0426-0.1579 | 0.083865 | 0.0-0.234 | 0.083252 | 0.63 |
|  | Caulimoviridae | 0.0-0.0736 | 0.040206 | 0.0-0.0596 | 0.030799 | 0.295 |
|  | Cilevirus | 0.0-0.0741 | 0.014669 | 0.0-0.0225 | 0.00785 | 0.244 |
|  | Circoviridae | 0.0-0.06 | 0.024367 | 0.0-3.125 | 0.364583 | 1 |
|  | Closteroviridae | 0.149-0.2773 | 0.219864 | 0.0-0.2935 | 0.170484 | 0.412 |
|  | Coronaviridae | 0.335-1.061 | 0.569445 | 0.0-0.985 | 0.513558 | 0.515 |
|  | Cystoviridae | 0.0-0.046 | 0.025874 | 0.0-0.0464 | 0.023278 | 0.932 |
|  | Dicistroviridae | 0.0-0.076 | 0.049746 | 0.0-0.0828 | 0.042442 | 0.551 |
|  | Endornaviridae | 0.669-0.1484 | 0.101847 | 0.0-0.154 | 0.08822 | 0.843 |
|  | Filoviridae | 0.0-0.089 | 0.030873 | 0.0-0.0511 | 0.021311 | 0.281 |
|  | Flaviviridae | 1.0-2.487 | 1.812658 | 0.0-9.375 | 2.609079 | 0.637 |
|  | Fuselloviridae | 0.164 | 0.2335 | 0.0-0.3 | 0.199854 | 0.799 |
|  | Geminiviridae | 0.074-0.504 | 0.277711 | 0.0-0.476 | 0.24269 | 0.755 |
|  | Hepadnaviridae | 0.0-0.0562 | 0.022592 | 0.0-0.0428 | 0.015407 | 0.345 |
|  | Herpesviridae | 5.185-9.192 | 6.833653 | 3.78-12.5 | 7.990006 | 0.187 |
|  | Idaeovirus | 0.0-0.0071 | 0.002261 | 0.0-0.00145 | 0.001637 | 0.604 |
|  | Iflaviridae | 0.0-0.0745 | 0.026047 | 0.0-0.0726 | 0.025494 | 0.82 |
|  | Inoviridae | 0.0667-0.144 | 0.114031 | 0.0-0.757 | 0.175893 | 1 |
|  | Iridoviridae | 4.041-6.78 | 4.862381 | 0.0-6.063 | 3.741993 | 0.152 |
|  | Lipothrixviridae | 0.298-0.49 | 0.404532 | 0.0-0.497 | 0.337193 | 0.932 |
|  | Luteoviridae | 0.076-0.295 | 0.152333 | 0.0-0.356 | 0.164994 | 0.977 |
|  | Marnaviridae | 0.0-0.029 | 0.013643 | 0.0-0.023 | 0.01101 | 0.629 |
|  | Microviridae | 0.0-0.0236 | 0.012201 | 0.0-1.515 | 0.177088 | 0.843 |
|  | Myoviridae | 23.311-29.39 | 26.49912 | 18.75-30.325 | 25.91827 | 0.276 |
|  | Nanoviridae | 0.0-0.0286 | 0.013645 | 0.0-0.757 | 0.095065 | 0.955 |
|  | Narnaviridae | 0.0-0.0476 | 0.015737 | 0.0-0.0585 | 0.01544 | 0.799 |
|  | Nodaviridae | 0.00557-0.03 | 0.020593 | 0.0-0.0347 | 0.021198 | 0.713 |
|  | Ophioviridae | 0.0-0.034 | 0.019884 | 0.0-0.031 | 0.015281 | 0.571 |
|  | Orthomyxoviridae | 0.0-0.0311 | 0.012842 | 0.0-0.0327 | 0.016718 | 0.477 |
|  | Papillomaviridae | 0.356-0.875 | 0.528308 | 0.0-1,515 | 0.669571 | 0.187 |
|  | Paramyxoviridae | 0.11-0.328 | 0.185567 | 0.0-0.337 | 0.186557 | 0.671 |
|  | Partitiviridae | 0.0-0.046 | 0.025464 | 0.0-0.0415 | 0.024303 | 0.977 |
|  | Parvoviridae | 0.0-0.223 | 0.119628 | 0.0-0.173 | 0.107141 | 0.932 |
|  | Phycodnaviridae | 3.988-7.19 | 6.701876 | 0.0-9.375 | 6.345966 | 0.522 |
|  | Picornaviridae | 0.224-0.381 | 0.319685 | 0.0-3.125 | 0.598421 | 0.89 |
|  | Polydnaviridae | 0.0-0.134 | 0.089013 | 0.0-3.125 | 0.445466 | 0.174 |
|  | Potyviridae | 0.305-1.267 | 0.56551 | 0.0-9.858 | 0.478892 | 0.799 |
|  | Poxviridae | 4.43-8.455 | 6.375315 | 0.0-7.874 | 5.887271 | 0.487 |
|  | Reoviridae | 0.149-0.36 | 0.263772 | 0.0-0.757 | 0.297337 | 0.978 |
|  | Retroviridae | 0.63-7.53 | 1.817353 | 0.0-2.267 | 1.440428 | 0.671 |
|  | Rhabdoviridae | 0.04-0.186 | 0.116365 | 0.0-0.205 | 0.111188 | 0.887 |
|  | Satellites | 0.0-0.0229 | 0.00833 | 0.0-0.0415 | 0.011288 | 0.977 |
|  | Secoviridae | 0.134-0.633 | 0.314 | 0.0-0.586 | 0.305044 | 0.887 |
|  | Siphoviridae | 16.735-24.393 | 20.80082 | 17.838-36.363 | 22.23387 | 0.89 |
|  | Sobemovirus | 0.0-0.0785 | 0.047197 | 0.0-0.0878 | 0.04986 | 0.63 |
|  | Tenuivirus | 0.0-0.0148 | 0.005356 | 0.0-0.0112 | 0.00374 | 0.469 |
|  | Togaviridae | 0.329-0.633 | 0.490424 | 0.0-0.757 | 0.484762 | 0.677 |
|  | Tombusviridae | 0.0-0.1038 | 0.037896 | 0.0-0.069 | 0.02945 | 0.496 |
|  | Totiviridae | 0.0-0.074 | 0.017908 | 0.0-0.0235 | 0.010408 | 0.257 |
|  | Tymoviridae | 0.038-0.223 | 0.098612 | 0.0-0.268 | 0.093991 | 0.27 |
|  | Umbravirus | 0.0-0.014 | 0.00524 | 0.0-0.0157 | 0.007705 | 0.345 |
|  | unclassified | 9.9-14.27 | 11.63036 | 7.575-12.533 | 11.17304 | 0.4377 |
